# Malaria: The Past and the Present

**DOI:** 10.3390/microorganisms7060179

**Published:** 2019-06-21

**Authors:** Jasminka Talapko, Ivana Škrlec, Tamara Alebić, Melita Jukić, Aleksandar Včev

**Affiliations:** 1 Faculty of Dental Medicine and Health, Josip Juraj Strossmayer University of Osijek, Crkvena 21, HR-31000 Osijek, Croatia; jtalapko@fdmz.hr (J.T.); avcev@fdmz.hr (A.V.); 2 Faculty of Medicine, Josip Juraj Strossmayer University of Osijek, Josipa Huttlera 4, HR-31000 Osijek, Croatia; tamara.alebic@gmail.com (T.A.); mjuki17@gmail.com (M.J.); 3 General Hospital Vukovar, Županijska 35, HR-32000 Vukovar, Croatia

**Keywords:** *Anopheles*, antimalarials, malaria, *Plasmodium*

## Abstract

Malaria is a severe disease caused by parasites of the genus *Plasmodium*, which is transmitted to humans by a bite of an infected female mosquito of the species *Anopheles*. Malaria remains the leading cause of mortality around the world, and early diagnosis and fast-acting treatment prevent unwanted outcomes. It is the most common disease in Africa and some countries of Asia, while in the developed world malaria occurs as imported from endemic areas. The sweet sagewort plant was used as early as the second century BC to treat malaria fever in China. Much later, quinine started being used as an antimalaria drug. A global battle against malaria started in 1955, and Croatia declared 1964 to be the year of eradication of malaria. The World Health Organization carries out a malaria control program on a global scale, focusing on local strengthening of primary health care, early diagnosis of the disease, timely treatment, and disease prevention. Globally, the burden of malaria is lower than ten years ago. However, in the last few years, there has been an increase in the number of malaria cases around the world. It is moving towards targets established by the WHO, but that progress has slowed down.

## 1. Introduction

Malaria affected an estimated 219 million people causing 435,000 deaths in 2017 globally. This burden of morbidity and mortality is a result of more than a century of global effort and research aimed at improving the prevention, diagnosis, and treatment of malaria [1]. Malaria is the most common disease in Africa and some countries in Asia with the highest number of indigenous cases. The malaria mortality rate globally ranges from 0.3–2.2%, and in cases of severe forms of malaria in regions with tropical climate from 11–30% [2]. Different studies showed that the prevalence of malaria parasite infection has increased since 2015 [3,4].

The causative agent of malaria is a small protozoon belonging to the group of *Plasmodium* species, and it consists of several subspecies. Some of the *Plasmodium* species cause disease in human [2,5]. The genus *Plasmodium* is an amoeboid intracellular parasite which accumulates malaria pigment (an insoluble metabolite of hemoglobin). Parasites on different vertebrates; some in red blood cells, and some in tissue. Of the 172 of *Plasmodium* species, five species can infect humans. These are *P. malariae*, *P.falciparum*, *P.vivax*, *P.ovale*, and *P.knowlesi*. In South-East Asia, the zoonotic malaria *P.knowlesi* is recorded. Other species rarely infect humans [5,6,7,8]. All the mentioned *Plasmodium* species cause the disease commonly known as malaria (Latin for *Malus aer*—bad air). Likewise, all species have similar morphology and biology [9].

The *Plasmodium* life cycle is very complex and takes place in two phases; sexual and asexual, the vector mosquitoes and the vertebrate hosts. In the vectors, mosquitoes, the sexual phase of the parasite’s life cycle occurs. The asexual phase of the life cycle occurs in humans, the intermediate host for malaria [9,10]. Human malaria is transmitted only by female mosquitoes of the genus *Anopheles*. The parasite, in the form of sporozoite, after a bite by an infected female mosquito, enters the human blood and after half an hour of blood circulation, enters the hepatocytes [11]. The first phase of *Plasmodium* asexual development occurs in the hepatocytes, and then in the erythrocytes. All *Plasmodium* species lead to the rupture of erythrocytes [7,9,12,13].

The most common species in the Americas and Europe are *P.vivax* and *P.malariae*, while in Africa it is *P.falciparum* [14].

## 2. Discovery of Malaria

It is believed that the history of malaria outbreaks goes back to the beginnings of civilization. It is the most widespread disease due to which many people have lost lives and is even thought to have been the cause of major military defeats, as well as the disappearance of some nations [15]. The first descriptions of malaria are found in ancient Chinese medical records of 2700 BC, and 1200 years later in the Ebers Papyrus [2]. The military leader Alexander the Great died from malaria [15]. The evidence that this disease was present within all layers of society is in the fact that Christopher Columbus, Albrecht Dürer, Cesare Borgia, and George Washington all suffered from it [16,17].

Although the ancient people frequently faced malaria and its symptoms, the fever that would occur in patients was attributed to various supernatural forces and angry divinities. It is, thus, stated that the Assyrian-Babylonian deity Nergal was portrayed as a stylized two-winged insect, as was the Canaan Zebub (‘Beelzebub, in translation: the master of the fly’) [17]. In the 4th century BC, Hippocrates described this disease in a way that completely rejected its demonic origins and linked it with evaporation from swamps which, when inhaled, caused the disease. That interpretation was maintained until 1880 and Laveran’s discovery of the cause of the disease [18]. Laveran, a French military surgeon, first observed parasites in the blood of malaria patients, and for that discovery he received the Nobel Prize in 1907 [19].

Cartwright and Biddis state that malaria is considered to be the most widespread African disease [14]. The causative agent of malaria is a small protozoon belonging to the group of *Plasmodium* species, and it consists of several subspecies [14].

## 3. The Development of Diagnostic Tests for Proving Malaria through History

Malaria can last for three and up to five years, if left untreated, and depending on the cause, may recrudesce. In *P. vivax* and *ovale* infections, the persistence of the merozoites in the blood or hypnozoites in hepatocytes can cause relapse months or years after the initial infection. Additionally, relapse of vivax malaria is common after *P. falciparum* infection in Southeast Asia. Relapse cases were observed in *P. falciparum* infections, which can lead to a rapid high parasitemia with subsequent destruction of erythrocytes [20,21]. Children, pregnant women, immunocompromised and splenectomized patients are especially vulnerable to malaria infection, as well as healthy people without prior contact with *Plasmodium*. A laboratory test for malaria should always confirm clinical findings. The proving of malaria is carried out by direct methods such as evidence of parasites or parts of parasites, and indirect methods that prove the antibodies to the causative agents (Table 1) [2,5,22].

The gold standard method for malaria diagnosis is light microscopy of stained blood films by Giemsa. Due to a lack of proper staining material and trained technicians, this method is not available in many parts of sub-Saharan Africa. The sensitivity of the method depends on the professional expertise, and it is possible to detect an infection with 10–100 parasites/μL of blood. A negative finding in patients with symptoms does not exclude malaria, but smears should be repeated three times in intervals of 12–24 h if the disease is still suspected [23,24]. Diagnosis of malaria using serologic testing has traditionally been done by immunofluorescence antibody testing (IFA). IFA is time-consuming and subjective. It is valuable in epidemiological studies, for screening possible blood donors. It also demands fluorescence microscopy and qualified technicians [23,25,26].

Rapid Diagnostic Tests (RDT) for the detection of antigens in the blood are immunochromatographic tests to prove the presence of parasite antigens. No electrical equipment, and no special experience or skills are required to perform these tests. The RDTs are now recommended by WHO as the first choice of test all across the world in all malaria-endemic areas. The sensitivity of the antigen test varies depending on the selected antigens represented in the test. For some RDTs is 50–100 parasites/μL (PfHRP2) to <100 parasites/μL [27,28]. The FDA approved the first RDT test in 2007. It is recommended that the results of all RDT tests should be confirmed by microscopic blood analysis [29]. It is known that antigens detected with RDT test remain in the blood after antimalarial treatment, but the existence of these antigens varies after treatment. The false-positive rates should be less than 10% [30]. Several RDT tests in the eight rounds of testing revealed malaria at a low-density parasite (200 parasites/μL), had low false-positive rates and could detect *P. falciparum* or *P. vivax* infections or both [30]. False-positive rates of *P. vivax* were typically small, between 5% and 15%. On the other hand, the false-positive rates of *P. falciparum* range from 3–32% [30,31]. Good RDTs might occasionally give false-negative results if the parasite density is low, or if variations in the production of parasite antigen reduce the ability of the RDT to detect the parasite. False negative results of the RDT test for *P. falciparum* ranged between 1% and 11% [31,32,33,34]. The overall sensitivity of RDTs is 82% (range 81–99%), and specificity is 89% (range 88–99%) [35].

Polymerase chain reaction (PCR) is another method in the detection of malaria. This method is more sensitive and more specific than all conventional methods in the detection of malaria. It can detect below one parasite/μL. PCR test confirms the presence of parasitic nucleic acid [23,36]. PCR results are often not available fast enough to be useful in malaria diagnosis in endemic areas. However, this method is most helpful in identifying *Plasmodium* species after diagnosis by microscopy or RDT test in laboratories that might not have microscopic experts. Additionally, PCR is useful for the monitoring of patients receiving antimalaria treatment [36,37].

Indirect methods are used to demonstrate antibodies to malaria-causing agents. Such methods are used in testing people who have been or might be at risk of malaria, such as blood donors and pregnant women. The method is based on an indirect immunofluorescence assay (IFA) or an ELISA test. The IFA is specific and sensitive but not suitable for a large number of samples, and the results are subjective evaluations. For serological testing, ELISA tests are more commonly used [26].

Rapid and accurate diagnosis of malaria is an integral part of appropriate treatment for affected person and the prevention of the further spread of the infection in the community.

## 4. Malaria Treatment through History

Already in the 2nd century BC, a sweet sagewort plant named Qinghai (Latin *Artemisia annua*) was used for the treatment of malaria in China [38]. Much later, in the 16th century, the Spanish invaders in Peru took over the cinchona medication against malaria obtained from the bark of the Cinchona tree (Latin *Cinchona succirubra*). From this plant in 1820 the French chemists, Pierre Joseph Pelletie, and Joseph Bienaimé Caventou isolated the active ingredient quinine, which had been used for many years in the chemoprophylaxis and treatment of malaria. In 1970, a group of Chinese scientists led by Dr. Youyou Tu isolated the active substance artemisinin from the plant *Artemisia annua*, an antimalarial that has proved to be very useful in treating malaria. For that discovery, Youyou Tu received the Nobel Prize for Physiology and Medicine in 2015 [39,40,41]. Most of the artemisinin-related drugs used today are prodrugs, which are activated by hydrolysis to the metabolite dihydroartemisinin. Artemisinin drugs exhibit its antimalarial activity by forming the radical via a peroxide linkage [42]. WHO recommends the use of artemisinin-based combination therapies (ACT) to ensure a high cure rate of *P. falciparum* malaria and reduce the spread of drug resistance. ACT therapies are used due to high resistance to chloroquine, sulfadoxine-pyrimethamine, and amodiaquine [1]. Due to the unique structure of artemisinins, there is much space for further research. Extensive efforts are devoted to clarification of drug targets and mechanisms of action, the improvement of pharmacokinetic properties, and identifying a new generation of artemisinins against resistant *Plasmodium* strains [42].

The German chemist Othmer Zeidler synthesized dichlorodiphenyltrichloroethane (DDT) in 1874 during his Ph.D. At that time, no uses of DDT was found, and it just became a useless chemical [43]. The insecticide property of DDT was discovered in 1939 by Paul Müller in Switzerland. DDT began to be used to control malaria at the end of the Second World War [40]. During the Second World War, the success of DDT quickly led to the introduction of other chlorinated hydrocarbons which were used in large amounts for the control of diseases transmitted by mosquito [43]. From the late Middle Ages until 1940, when DDT began to be applied, two-thirds of the world’s population had been exposed to malaria, a fact that represented a severe health, demographic, and economic problem [29,40,41,44,45]. DDT is an organochlorine pesticide which was applied in liquid and powder form against the insects. During the Second World War people were sprayed with DDT. After the war, DDT became a powerful way of fighting malaria by attacking the vector [43].

Five Nobel Prizes associated with malaria were awarded: Youyou Tu in 2015. Ronald Ross received the Nobel Prize in 1902 for the discovery and significance of mosquitoes in the biology of the causative agents in malaria. In 1907, the Nobel was awarded to the already-mentioned Charles Louis Alphonse Laveran for the discovery of the causative agent. Julius Wagner-Jauregg received it in 1927 for the induction of malaria as a pyrotherapy procedure in the treatment of paralytic dementia. In 1947 Paul Müller received it for the synthetic pesticide formula dichlorodiphenyltrichloroethane.

Attempts to produce an effective antimalarial vaccine and its clinical trials are underway. Over the past several decades’ numerous efforts have been made to develop effective and affordable preventive antimalaria vaccines. Numerous clinical trials are completed in the past few years. Nowadays are ongoing clinical trials for the development of next-generation malaria vaccines. The main issue is *P. vivax* vaccine, whose research requires further investigations to identify novel vaccine candidates [46,47,48]. Despite decades of research in vaccine development, an effective antimalaria vaccine has not yet been developed (i.e., with efficacy higher than 50%) [49,50,51]. The European Union Clinical Trials Register currently displays 48 clinical trials with a EudraCT protocol for malaria, of which 13 are still ongoing clinical trials [52]. The malaria parasite is a complex organism with a complex life cycle which can avoid the immune system, making it very difficult to create a vaccine. During the different stages of the *Plasmodium* life cycle, it undergoes morphological changes and exhibits antigenic variations. *Plasmodium* proteins are highly polymorphic, and its functions are redundant. Also, the development of malaria disease depends on the *Plasmodium* species. That way, a combination of different adjuvants type into antigen-specific formulations would achieve a higher efficacy [53,54]. Drugs that underwent clinical trials proved to be mostly ineffective [5,7,55]. However, many scientists around the world are working on the development of an effective vaccine [56,57,58]. Since other methods of suppressing malaria, including medication, insecticides, and bed nets treated with pesticides, have failed to eradicate the disease, and the search for a vaccine is considered to be one of the most important research projects in public health by World Health Organization (WHO).

The best way to fight malaria is to prevent insect bites. Malaria therapy is administered using antimalarial drugs that have evolved from quinine. According to its primary effect, malarial vaccines are divided into pre-erythrocytic (sporozoite and liver-stage), blood-stage, and transmission-blocking vaccines [9,54]. Most medications used in the treatment are active against parasitic forms in the blood (the type that causes disease) (Table 2) [59]. The two crucial antimalarial medications currently used are derived from plants whose medical importance has been known for centuries: artemisinin from the plant Qinghao (*Artemisia annua* L, China, 4th century) and quinine from *Cinchona* (South America, 17th century). Side-by-side with artemisinin, quinine is one of the most effective antimalarial drugs available today [13,39,40]. Doxycycline is indicated for malaria chemoprophylaxis for travel in endemic areas. It is also used in combination with the quinine or artesunate for malaria treatment when ACT is unavailable or when the treatment of severe malaria with artesunate fails. The disadvantage of doxycycline is that children and pregnant women cannot use it [29]. Due to the global resistance of *P. falciparum* to chloroquine, ACTs are recommended for the treatment of malaria, except in the first trimester of pregnancy. ACTs consist of a combination of an artemisinin derivative that fast decreases parasitemia and a partner drug that eliminates remaining parasites over a more extended period. The most common ACTs in use are artemether-lumefantrine, artesunate-amodiaquine, dihydroartemisinin-piperaquine, artesunate-mefloquine, and artesunate with sulfadoxine-pyrimethamine. The ACTs were very efficient against all *P. falciparum* until recently when numbers of treatment failures raised in parts of Southeast Asia. Atovaquone-proguanil is an option non-artemisinin-based treatment that is helpful for individual cases which have failed therapy with usual ACTs. Although, it is not approved for comprehensive implementation in endemic countries because of the ability for the rapid development of atovaquone resistance. Quinine remains efficient, although it needs a long course of treatment, is poorly tolerated, especially by children, and must be combined with another drug, such as doxycycline or clindamycin. Uncomplicated vivax, malariae, and ovale malaria are handled with chloroquine except in case of chloroquine-resistant *P. vivax* when an ACT is used [7,29,60,61,62].

### 4.1. Malaria in Europe

In Europe, malaria outbreaks occurred in the Roman Empire [63,64] and the 17th century. Up until the 17th century it was treated as any fever that people of the time encountered. The methods applied were not sufficient and included the release of blood, starvation, and body cleansing. As the first effective antimalarial drug, the medicinal bark of the Cinchona tree containing quinine was mentioned and was initially used by the Peruvian population [14]. It is believed that in the fourth decade of the 17th century it was transferred to Europe through the Spanish Jesuit missionaries who spread the treatment to Europe [65].

Contemporary knowledge of malaria treatment is the result of the work of a few researchers. Some of researchers are Alphonse Laveran, Ronald Ross, and Giovanni Battista Grassi. In November 1880, Laveran, who worked as a military doctor in Algeria, discovered the causative agents of malaria in the blood of mosquitoes and found that it was a kind of protozoa [66]. Laveran noticed that protozoa could, just like bacteria, live a parasitic way of life within humans and thus cause disease [66]. Nearly two decades later, more precisely in 1898, Ronald Ross, a military doctor in India, discovered the transmission of bird malaria in the saliva of infected mosquitos, while the Italian physician Giovanni Battista Grassi proved that malaria was transmitted from mosquitoes to humans, in the same year. He also proved that not all mosquitoes transmit malaria, but only a specific species (*Anopheles*) [17]. This discovery paved the way for further research.

The global battle against malaria started in 1955, and the program was based on the elimination of mosquitoes using DDT and included malarial areas of the United States, Southern Europe, the Caribbean, South Asia, but only three African countries (South Africa, Zimbabwe, and Swaziland). In 1975, the WHO announced that malaria had been eradicated in Europe and all recorded cases were introduced through migration [67,68].

### 4.2. Malaria in Croatia

In Croatia, the first written document that testifies to the prevention of malaria is the Statute of the town of Korčula from 1265. In 1874, the Law on Health Care of Croatia and Slavonia established the public health service that was directed towards treating malaria. There was no awareness nor proper medical knowledge about malaria, but the drainage was carried out to bring the ‘healthy air’ in the cities [69,70]. In 1798 physician Giuseppe Arduino notified the Austrian government about malaria in Istria. A government representative Vincenzo Benini accepted a proposed sanitary measure of the drainage of wetlands [71]. In 1864, the drainage of wetlands around Pula and on the coastal islands began, and since 1902 a program for the suppression of malaria by treatment of patients using quinine has been applied [72]. In 1922, the Institute for Malaria was founded in Trogir. In 1923, on the island of Krk, a project was started to eradicate malaria by the sanitation of water surfaces and the treatment of the patients with quinine, led by Dr. Otmar Trausmiller [66]. Since 1924, besides chemical treatment, biological control of mosquitoes has been established by introducing the fish *Gambusia holbrooki* to Istria and the coast [73]. In 1930 legislation was passed to enforce village sanitation, which included the construction of water infrastructure and safe wells, contributing to the prevention of malaria. Regular mosquito fogging with arsenic green (copper acetoarsenite) was introduced, and larvicidal disinfection of stagnant water was carried out.

Since malaria occurs near swamps, streams, ravines, and places where mosquitoes live near water, this disease has been present throughout history in Croatia, and it has often become an epidemic [74]. It was widespread in the area of Dalmatia, the Croatian Littoral region, Istria, and river flows. In the area of the Croatian Littoral, it was widespread on some islands, such as Krk, Rab, and Pag, while the mainland was left mainly clear of it [75]. The ethnographer Alberto Fortis (1741–1803) who traveled to Dalmatia, noted impressions recording details of malaria that was a problem in the Neretva River valley. Fortis wanted to visit that area, but the sailors on ship were afraid, probably because the were afraid to go to a place where there had been a disease outbreak known as the Neretva plague [76]. This Neretva plague was, in fact, malaria, and it is believed that due to it, the Neretva was nicknamed “Neretva—damned by God” [77,78]. Speaking of the Neretva region, Fortis states that the number of mosquitoes in that wetland area was so high that people had to sleep in stuffy canopy tents to defend themselves. Fortis also states that there were so many mosquitoes that he was affected by it. During the stay, Fortis met a priest who had a bump on the head claiming it had occurred after a mosquito bite and believed that the fever that infected the people of the Neretva Valley was also a consequence of the insect bites there [76]. In a manuscript, Dugački described some of the epidemics in Croatia. Thus, noted the small population of Nin in 1348, which was the result of the unhealthy air and high mortality of the population. Three centuries later, in 1646, the fever was mentioned in Novigrad, while the year 1717 was crucial for to the Istrian town of Dvigrad, which was utterly deserted due to malaria. At the beginning of the 20th century, more precisely in 1902, the daily press reported that the Provincial Hospital in Zadar was full of people affected by malaria. The extent to which this illness was widespread is proved by the fact that at the beginning of the 20th century about 180,000 people suffered from it in Dalmatia [18]. The volume and frequency of epidemics in Dalmatia resulted in the arrival of the Italian malariologist Grassi and the German parasitologist Schaudin. The procedures of quininization began to be applied, and in 1908 25 physicians and 423 pill distributors were to visit the villages and divide pills that had to be taken regularly to the people to eradicate malaria [75].

Likewise, in Slavonia, malaria had also a noticeable effect, and it was widespread in the 18th century due to a large number of swamps that covered the region. Such areas were extremely devastating for settlers who were more vulnerable to the disease than its domestic population [79]. Friedrich Wilhelm von Taube (1728–1778) recorded the disease and stated that the immigrant Germans were primarily affected by malaria and that the cities of Osijek and Petrovaradin can be nicknamed "German Cemeteries" [80]. According to Skenderović, the high mortality of German settlers from malaria was not limited only to the Slavonia region but also to the Danubian regions in which the Germans had settled in the 18th century, with Banat and Bačka [79] having the most significant number of malaria cases. The perception of Slavonia in the 18th century was not a positive one. Even Taube stated that Slavonia was not in good standing in the Habsburg Monarchy and that the nobility avoided living there. As some of the reasons for this avoidance, Taube mentioned the unhealthy air and the many swamps in the area around in which there was a multitude of insects. Taube noted that mosquitoes appear to be larger than in Germany and that its bite was much more painful. A change in the situation could only be brought about by drying the swamp, in his opinion [80]. Since malaria had led to the death of a large number of people, the solution had to be found to stop its further spread. Swamp drying was finally accepted by the Habsburg Monarchy and some European countries as a practical solution and, thus, its drainage began during the 18th century, resulting in cultivated fields [79].

Since epidemics of malaria continued to occur, there is one more significant record of the disease in the Medical Journal of 1877. In it, the physician A. Holzer cites his experiences from Lipik and Daruvar where he had been a spa physician for a long time. Holzer warns of the painful illness noticed at spa visitors suffering from the most in July and August. As a physician, Holzer could not remain indifferent to the fact that he did not see anyone looking healthy. It also pointed out that other parts of Croatia were not an exception. As an example, Holzer noted Virovitica County, where malaria was also widespread. He wanted to prevent the development and spread of the illness. Believing that preventing the toxic substances from rising into the air would stop the disease, the solution was to use charcoal that has the properties of absorbing various gases and, thus, prevents vapor rising from the ground [81].

Dr. Andrija Štampar (1888–1958) holds a prominent place in preventing the spread of malaria. Štampar founded the Department of Malaria, and numerous antimalaria stations, hygiene institutes, and homes of national health. Dr. Štampar devoted his life to educating the broader population about healthy habits and, thus, prevents the spread of infectious diseases. Many films were shown, including a film entitled ‘Malaria of Trogir’ in Osijek in 1927, with numerous health lectures on malaria [82]. After the end of the Second World War, a proposal for malaria eradication measures was drafted by Dr. Branko Richter. These measures, thanks to Dr. Andrija Štampar, are being used in many malaria-burdened countries. For the eradication of malaria in Croatia and throughout Yugoslavia, DDT has been used since 1947 [83].

Malaria is still one of the most infectious diseases that cause far more deaths than all parasitic diseases together. Malaria was eradicated in Europe in 1975. After that year, malaria cases in Europe are linked to travel and immigrants coming from endemic areas. Although the potential for malaria spreading in Europe is very low, especially in its western and northern parts, it is still necessary to raise awareness of this disease and keep public health at a high level in order to prevent the possibility of transmitting the disease to the most vulnerable parts of Europe [84].

**Figure 1:**
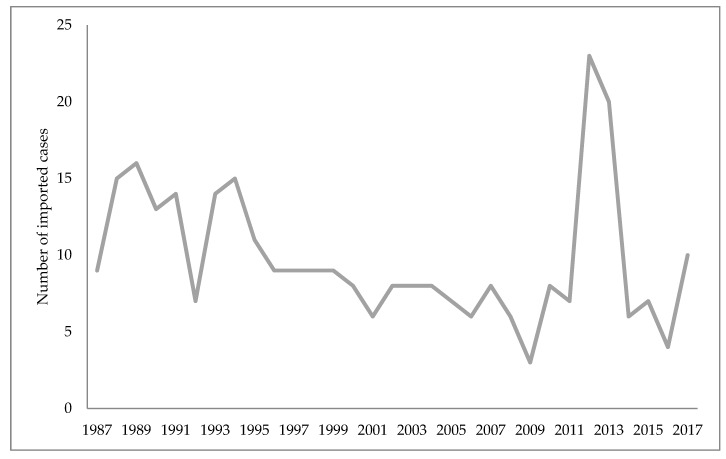
Imported malaria cases in Croatia from 1987–2017.

**Figure 2:**
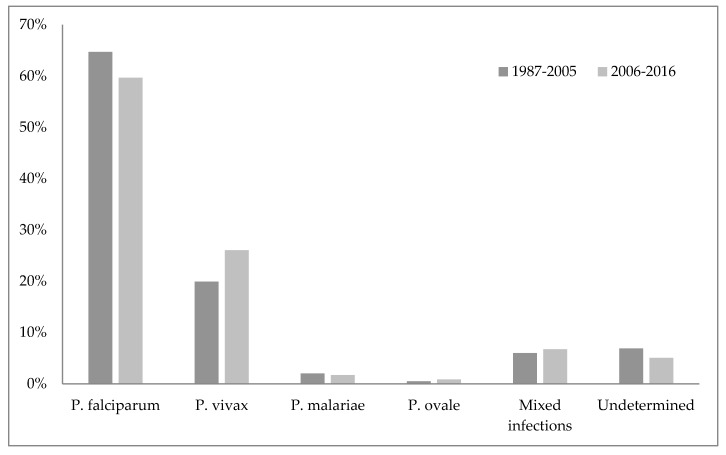
The causative agents of imported malaria in Croatia.

Unofficial data show that malaria disappeared from Croatia in 1958, while the World Health Organization cites 1964 as the year when malaria was officially eradicated in Croatia [45,75]. Nonetheless, some cases of imported malaria have been reported in Croatia since 1964. The imported malaria is evident concerning Croatia’s orientation to maritime affairs, tourism, and business trips. Namely, malaria is introduced to Croatia by foreign and domestic sailors, and in rare cases by tourists, mainly from the countries of Africa and Asia [75,85]. According to the reports of the Croatian Institute of Public Health, since the eradication of this disease 423 malaria cases have been reported, all imported [86]. Figure 1 shows the number of imported malaria cases in Croatia from 1987–2017, and Figure 2 the causative *Plasmodium* species of those cases (Figure 1 and Figure 2) [86,87].

There is also massive and uncontrollable migration from Africa and Asia (mostly due to climate change) of both humans and birds, from countries with confirmed epidemics. This issue is an insurmountable problem if measured by the traditional approach. Insecticides (DDT, malathion, etc.) synthetic pyrethroids, in addition to inefficiency, impact the environment (harm bees, fruits, vines, etc.). Consequently, scientists have patiently established a mosquito control strategy (University of Grenoble, Montpellier) which includes a meticulous solution to the mosquito vector effect (malaria, arbovirus infection, West Nile virus) by changes in agriculture, urbanism, public services hygiene [88].

Northeastern Slavonia is committed to applying methods that are consistent with such achievements, with varying success, as certain limitations apply to protected natural habitats (Kopački rit) [89].

There is a historical link between population movement and global public health. Due to its unique geostrategic position, in the past, Croatia has been the first to experience epidemics that came to Europe through land and sea routes from the east. Adriatic ports and international airports are still a potential entry for the import of individual cases of communicable diseases. Over the past few years, sailors, as well as soldiers who worked in countries with endemic malaria, played a significant role in importing malaria into Croatia. Successful malaria eradication has been carried out in Croatia. Despite that in Croatia are still many types of *Anopheles*, which means that the conditions of transmission of the imported malaria from the endemic areas still exist. The risk of malaria recrudesce is determined by the presence of the vector, but also by the number of infected people in the area. Due to climate change, it is necessary to monitor the vectors and people at risk of malaria. Naturally- and artificially-created catastrophes, such as wars and mass people migration from endemic areas, could favor recrudescing of malaria. Once achieved, eradication would be maintained if the vector capacities are low and prevention measures are implemented. The increased number of malaria cases worldwide, the recrudesce of indigenous malaria cases in the countries where the disease has been eradicated, the existence of mosquitoes that transmit malaria and the number of imported malaria cases in Croatia are alarming facts. Health surveillance, including obligatory and appropriate prophylaxis for travelers to endemic areas, remains a necessary public health care measure pointed at managing malaria in Croatia.

## 5. Malaria Trends in the World

**Figure 3:**
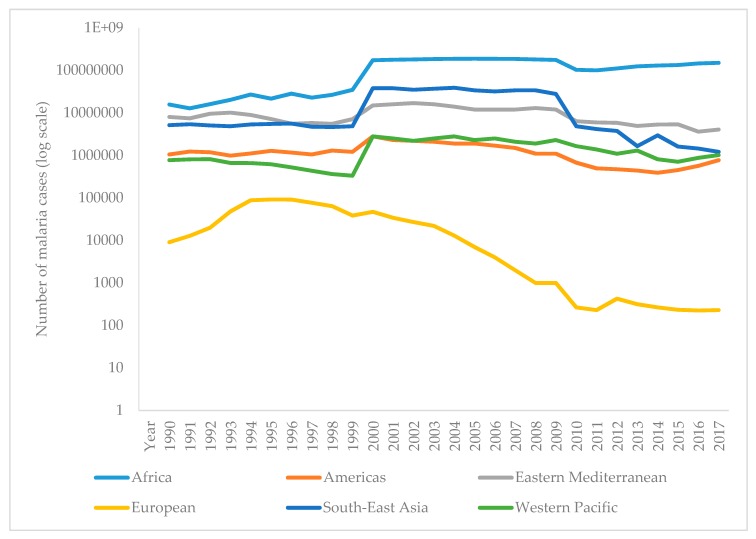
Reported malaria cases per WHO region from 1990–2017.

**Figure 4:**
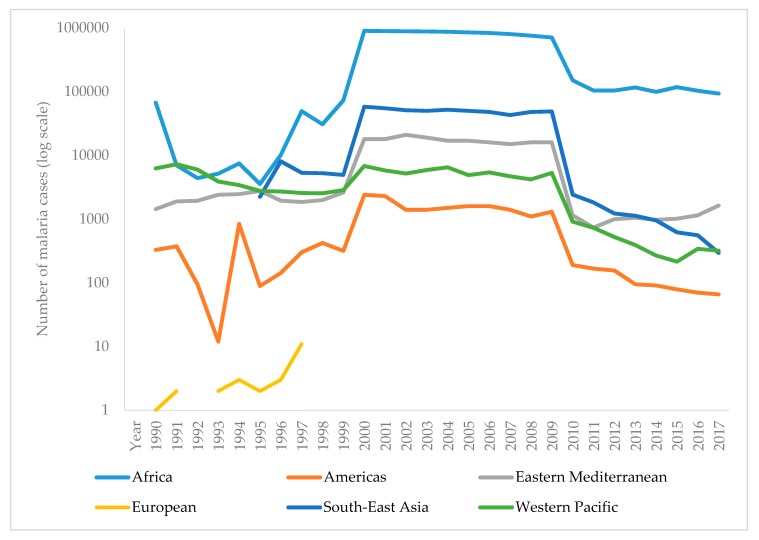
Reported malaria deaths per WHO region from 1990–2017.

The WHO report on malaria in 2017 shows that it is difficult to achieve two crucial goals of a Global Technical Strategy for Malaria. These are a reduction in mortality and morbidity by at least 40% by 2020. Since 2010, there has been a significant reduction in the burden of malaria, but analysis suggests a slowdown, and even an increase in the number of cases between 2015 and 2017. Thus, the number of malaria cases in 2017 has risen to 219 million, compared to 214 million cases in 2015 and 239 million cases in 2010. Figure 3 presents the reported number of malaria cases per WHO region from 1990–2017 [1,90]. The most critical step in the global eradication of malaria is to reduce the number of cases in countries with the highest burden (many in Africa). The number of deaths from disease is declining, thus, in 2017 there were 435,000 deaths from malaria globally, compared with 451,000 in 2016, and 607,000 deaths in 2010. Figure 4 presents the number of malaria deaths from 1990-2017 [1,90]. Despite the delay in global progress, there are countries with decreasing malaria cases during 2017. Thus, India in 2017, compared with 2016, recorded a 24% decline of malaria cases. The number of countries reporting less than 10,000 malaria cases is growing, from 37 countries in 2010, to 44 in 2016, and to 46 in 2017. Furthermore, the number of countries with fewer than 100 indigenous malaria cases growing from 15 in 2010, to 26 countries in 2017 [1].

Funding in malaria has not changed much. During 2017, US$3.1 billion was invested in malaria control and elimination globally. That was 47% of the expected amount by 2020. The USA was the largest single international donor for malaria in 2017 [1,91].

The most common global method of preventing malaria is insecticide-treated bed nets (ITNs). The WHO report on insecticide resistance showed that mosquitoes became resistant to the four most frequently used classes of insecticides (pyrethroids, organochlorines, carbamates, and organophosphates), which are widespread in all malaria-endemic countries [1,7,92].

Drug resistance is a severe global problem, but the immediate threat is low, and ACT remains an effective therapy in most malaria-endemic countries [1,93].

According to the WHO, Africa still has the highest burden of malaria cases, with 200 million cases (92%) in 2017, then Southeast Asia (5%), and the Eastern Mediterranean region (2%). The WHO Global Technical Strategy for Malaria by 2020 is the eradication of malaria from at least ten countries that were malaria-endemic in 2015 [1].

The march towards malaria eradication is uneven. Indigenous cases in Europe, Central Asia, and some countries in Latin America are now sporadic. However, in many sub-Saharan African countries, elimination of malaria is more complicated, and there are indications that progress in this direction has delayed. Elimination of vivax and human knowlesi malaria infections are another challenge [7].

## 6. Conclusions

The campaign to eradicate malaria began in the 1950s but failed globally due to problems involving the resistance of mosquitoes to the insecticides used, the resistance of malaria parasites to medication used in the treatment, and administrative issues. Additionally, the first eradication campaigns never included most of Africa, where malaria is the most common. Although the majority of forms of malaria are successfully treated with the existing antimalarials, morbidity and mortality caused by malaria are continually increasing. This issue is the consequence of the ever-increasing development of parasite resistance to drugs, but also the increased mosquito resistance to insecticides, and has become one of the most critical problems in controlling malaria over recent years. Resistance has been reported to all antimalarial drugs. Therefore, research into finding and testing new antimalarials, as well as a potential vaccine, is still ongoing, mainly due to the sudden mass migration of humans (birds, parasite disease vector insects) from areas with a large and diverse infestation.

The process towards eradication in some countries confirms that current tools could be sufficient to eradicate malaria. The spread of insecticide resistance among the vectors and the rising ACT failures indicate that eradication of malaria by existing means might not be enough.

Thus, given the already complicated problem of overseeing and preventing the spread of the disease, it will be necessary to supplement and change the principles, strategic control, and treatment of malaria.

## Figures and Tables

**Table 1:** Diagnostic tests for proving malaria.

	Advantages	Disadvantages
**Direct methods**		
Microscopic analysis	Fast test, cheap	Required much experience as well as equipment
Rapid diagnostic tests	Quick and simple	Less sensitive and accurate, price
Molecular tests	Correct determination of type, highly sensitive and accurate	Price, long-term in a large number of cases
**Indirect methods**		
Indirect immunofluorescence	Specific, sensitive	Long time to perform, subjective evaluation of results
ELISA	Correct determination of type, specific, sensitive	Long time to perform, price

**Table 2:** Overview of the most commonly used antimalarials.

Medication Name	Year of Discovery/Synthesis	Origin	Usage	Mechanism of Action	Side Effects	Advantages/Disadvantages
Quinine	1600	*Cinchona* tree, South America	Resistance to chloroquine, prophylaxis and treatment of malaria	Inhibition of DNA and RNA synthesis	Headache, abortion, or congenital malformations if taken during pregnancy	Toxic, less effective than other medication
Chloroquine	1934	Synthesized by German scientist Hans Andersag	Most powerful remedy for the prophylaxis and treatment of malaria	Inhibition of DNA and RNA synthesis	Gastrointestinal disturbances, headache, skin irritation	Developed resistance of most strains *of P. falciparum* to the medication
Primaquine	1953	The 8-aminoquinoline derivative	Infections with *P. vivax* and *P. ovale*, prophylaxis and treatment of malaria	Interferes in transport chain of electrons and destroys parasite mitochondria	Anorexia, nausea, anemia, headaches, contraindicated in pregnancy and children under 4 years of age	Prevent relapse in *P. vivax* and *P. ovale* infection
Doxycycline	1960	Pfizer Inc. New York	Prophylaxis in areas with chloroquine resistance and against mefloquine resistant *P. falciparum*	Inhibition of protein synthesis by binding to 30S ribosomal subunit	Gastrointestinal disorders, nausea, vomiting, photosensitivity	Effective and cheap, use for treatment and prophylaxis in all malarious areas
Mefloquine	1971	USA army and WHO	Multiresistant *P. falciparum* strains, prophylaxis and treatment of malaria	Damage to parasite membrane	Gastrointestinal disorders, CNS disorder, contraindicated in pregnancy and patients with epilepsy	Partial resistance, brain damage
Proguanil (chloroguanide)	1953	Biguanide derivate	Prophylaxis in infections with *P. falciparum*	Inhibition of DNA synthesis	Digestive problems only in large doses	The least toxic antimalarial drug
Pyrimethamine	1953	Pyrimidine derivatives	For tissue parasites, prophylaxis and treatment of malaria	Folic acid antagonist	Gastrointestinal disorders, neuropathy, in high doses also megaloblastic anemia	Rapid development of resistance
Atovaquone/proguanil	2000	Ubiquinone analog	For the prophylaxis and treatment of malaria	Inhibition of cytochrome bc1 in *Plasmodium*	Nausea, vomiting, diarrhea, headache, dizziness, anxiety, difficulty falling asleep, rash, fever	Most commonly used, fewer side effects and more expensive than mefloquine, *P. falciparum* resistance

CNS—central nervous system.

## Abbreviations

ACT	Artemisinin-based combination therapy
CNS	Central nervous system
DDT	Dichlorodiphenyltrichloroethane
ELISA	Enzyme-linked immunosorbent assay
FDA	Food and Drug Administration
IFA	Immunofluorescence test
ITN	Insecticide-treated bed nets
PCR	Polymerase chain reaction
PfHRP2	Plasmodium falciparum histidine-rich protein 2
RDT	Rapid diagnostic tests
WHO	World Health Organization
